# Frailty and risk of systemic atherosclerosis: A bidirectional Mendelian randomization study

**DOI:** 10.1371/journal.pone.0304300

**Published:** 2024-05-23

**Authors:** Liugang Xu, Yajun Wang, Hongyun Ji, Wei Du, Chunhui You, Jin Chen, Jianyu Jiang, Yisi Shan, Qian Pan, Ruihong Cao

**Affiliations:** 1 Department of Cardiology, Zhangjiagang Hospital of Traditional Chinese Medicine Affiliated to Nanjing University of Traditional Chinese Medicine, Zhangjiagang, Jiangsu, People’s Republic of China; 2 Department of Neurology, Zhangjiagang Hospital of Traditional Chinese Medicine Affiliated to Nanjing University of Traditional Chinese Medicine, Zhangjiagang, Jiangsu, People’s Republic of China; Tehran University of Medical Sciences, ISLAMIC REPUBLIC OF IRAN

## Abstract

**Background:**

Numerous observational studies have reported an association between frailty and atherosclerosis. However, the causal relationship between frailty and the occurrence of atherosclerosis in different anatomical sites remains unclear. we conducted a bidirectional Mendelian randomization (MR) study to evaluate the causal relationship between the frailty index (FI), and both systemic atherosclerosis and lipids.

**Methods:**

We obtained summary statistics from large-scale genome-wide association studies (GWAS) of various phenotypes, including frailty (n = 175,226), coronary atherosclerosis (n = 56,685), cerebral atherosclerosis (n = 150,765), peripheral arterial disease (PAD) (n = 361,194), atherosclerosis at other sites (n = 17,832), LDL-C (n = 201,678), HDL-C (n = 77,409), and triglycerides (n = 78,700). The primary MR analysis employed the inverse variance weighted (IVW) method. Furthermore, to assess reverse causality, we employed inverse MR and multivariate MR analysis.

**Results:**

Genetically predicted FI showed positive associations with the risk of coronary atherosclerosis (OR = 1.47, 95% CI 1.12–1.93) and cerebral atherosclerosis (OR = 1.99, 95% CI 1.05–3.78), with no significant association (*p* >0.05) applied to peripheral arterial disease and atherosclerosis at other sites. Genetically predicted FI was positively associated with the risk of triglycerides (OR = 1.31, 95% CI 1.08–1.59), negatively associated with the risk of LDL-C (OR = 0.87, 95% CI 0.78–0.97), and showed no significant association with the risk of HDL-C (*p* >0.05). Furthermore, both reverse MR and multivariate MR analyses demonstrated a correlation between systemic atherosclerosis, lipids, and increased FI.

**Conclusion:**

Our study elucidated that genetically predicted FI is associated with the risk of coronary atherosclerosis and cerebral atherosclerosis by the MR analysis method, and they have a bidirectional causal relationship. Moreover, genetically predicted FI was causally associated with triglyceride and LDL-C levels. Further understanding of this association is crucial for optimizing medical practice and care models specifically tailored to frail populations.

## Introduction

Frailty, an age-related physical state, manifests as impaired physiological function in multiple organs or systems [1]. It contributes to the aging process across various bodily systems [2], leading to an enduring state that heightens the risk of cardiovascular events, falls, and disability in response to stressors [2–4]. Frail populations often exhibit a range of chronic conditions, such as coronary heart disease, heart failure, and ischemic stroke, which can result in varying degrees of physical disability. Observational studies have consistently linked frailty with coronary heart disease [5,6], ischemic stroke [7,8], and peripheral arterial disease [9] (PAD), all of which are associated with the underlying pathology of atherosclerosis. However, observational studies are susceptible to confounding bias and reverse causal associations, and whether a bidirectional association exists between frailty and systemic atherosclerosis with lipids remains uncertain.

Mendelian randomization (MR) is a genetic epidemiological approach that relies on data from genome-wide association studies (GWAS). It utilizes independent genetic variants as instrumental variables to investigate causal relationships between an exposure and an outcome [10]. The fundamental principle behind MR is that an individual’s genotype is randomly assigned during gamete formation. This helps to eliminate the possibility of confounding bias and reverse causal associations [10,11]. As a result, MR can effectively reduce the impact of reverse causation and other confounding factors, thereby providing valuable insights into the underlying relationship between frailty and systemic atherosclerosis. This is a significant finding, with important implications for both the public and clinical sectors.

The frailty index (FI) is widely recognized as the preferred instrument for evaluating frailty [1,12]. It assigns a continuous score ranging from 0 (no deficits) to 1 (all deficits) based on the cumulative presence of health deficits, encompassing somatic symptoms, psychological factors, comorbidities, and disability [1]. A higher FI score is associated with various clinical outcomes, including a range of chronic conditions, disability, and mortality [13]. In our study, we employed a bidirectional MR approach to examine the causal relationship between frailty, as measured by the FI, and systemic atherosclerosis. We investigated different sites of atherosclerosis, including coronary atherosclerosis, cerebral atherosclerosis, PAD, and atherosclerosis at other sites. Additionally, we accounted for the assessment of lipids because lipid-driven intravascular plaques are the pathological basis of atherosclerosis.

## Materials and methods

We conducted a two-sample MR analysis using GWAS summary statistics to estimate the causal associations between FI and systemic atherosclerosis as well as lipids. We utilized GWAS summary statistics for frailty indices as the exposure variable and selected relevant single nucleotide polymorphisms (SNPs) as instrumental variables (IVs). Systemic atherosclerosis summary statistics were used as the outcome. Additionally, we investigated the causal association of FI with lipids, including LDL cholesterol (LDL-C), HDL cholesterol (HDL-C), and triglycerides. To explore the reverse direction of causality, we employed bidirectional MR analysis, and multivariate MR analysis was also used to estimate causal associations.

### Exposure sources

Summary data for the frailty index were retrieved from the most recent GWAS meta-analysis of the UK Biobank and TwinGene, we identified the frailty index as an exposure factor [14]. They measured frailty by the FI, which was based on the accumulation of 49 health deficits during the life course and has been well-validated and widely used in clinical practice [1]. The UK Biobank included a total of 164,610 participants aged 60 to 70 with European ancestry, including 84,819 females, with data from 49 self-reported deficits. The TwinGene study included 10,616 Swedish population samples, with ages ranging from 41 to 87, and females accounted for 5,577 individuals (52.5%). The complete GWAS summary statistics can be found in the GWAS catalog under study number GCST90020053 (https://www.ebi.ac.uk/gwas/search?query=GCST90020053).

### Outcome sources

The FinnGen study [15] integrated genomic data with healthcare information from the Finnish National Health Registry, utilizing the Finnish biobank that currently encompasses over 370,000 samples. Data on coronary atherosclerosis and atherosclerosis at other sites can be found (https://r9.finngen.fi/). Rainer Malik et al [7] conducted a study of over 520,000 European subjects in a GWAS meta-analysis examined the large artery atherosclerosis-related ischemic stroke subtype, which we defined as cerebral atherosclerosis, and relevant data are available in the Integrative Epidemiology Unit (IEU) OpenGWAS data infrastructure, study number: ebi-a-GCST005840. (https://gwas.mrcieu.ac.uk/datasets/ebi-a-GCST005840/).

GWAS data based on the UK Biobank study pooled information from over approximately 500,000 samples [16] and PAD data with 361,194 European samples are available in the Neale lab public database. The latest GWAS data for LDL-C, HDL-C, and Triglycerides are available in the MRC IEU OpenGWAS data infrastructure under study numbers: ieu-b-5089, ieu-b-4844, and ieu-b-4850. Data sources are summarized in Table 1.

**Table 1:** Overview of data sources.

Phenotype	Source	Region	Year	Sample size
FI	UK Biobank and TwinGene	European、Swedish	2021	175,226
Coronary arteriosclerosis	Finngen Research Project	Finnish	2021	56,685
Cerebral atherosclerosis	IEU Open Gwas	European	2018	150,765
Peripheral arterial disease	UK Biobank	European	2018	361,194
atherosclerosis of other sites	Finngen Research Project	Finnish	2021	17,832
LDL-C	IEU Open Gwas	European	2022	201,678
HDL-C	IEU Open Gwas	European	2022	77,409
Triglycerides	IEU Open Gwas	European	2022	78,700

FI, Frailty index; PAD, Peripheral arterial disease; LDL-C, Low-density lipoprotein cholesterol; HDL-C, High-density lipoprotein cholesterol.

### Selection of instrumental variables

**Fig 1:**
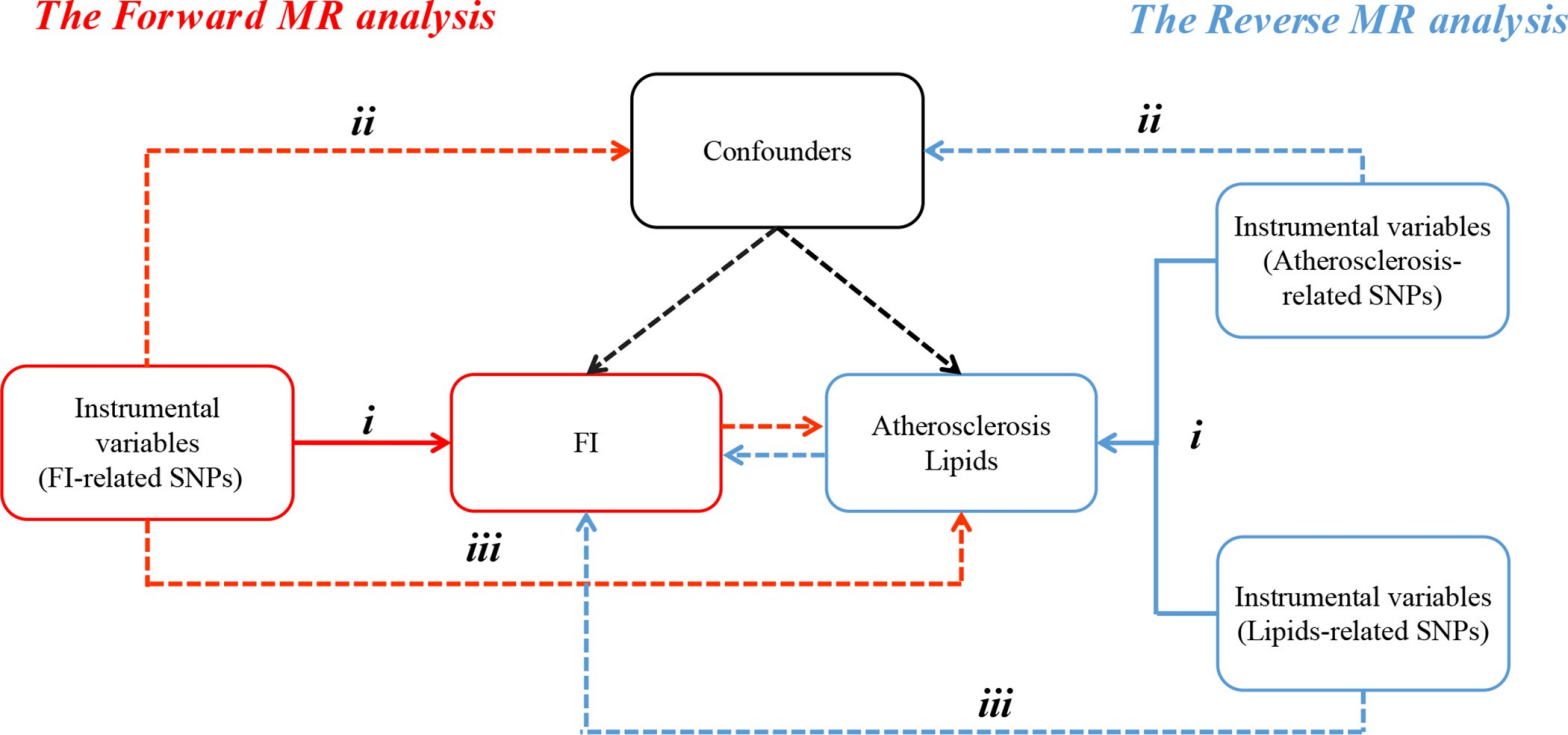
The study design. The MR analysis was based on three fundamental assumptions: The relevance assumption, independence assumption, and exclusivity hypothesis. Red represents the positive MR analysis pathway (FI-related). Blue represents the reverse MR analysis pathway (atherosclerosis-related and lipid-related). MR, Mendelian randomization; FI, Frailty index; SNP, Single nucleotide polymorphism.

The requirements of MR analysis for instrumental variables must satisfy three assumptions (Fig 1): (i) Relevance assumption: IVs must be correlated with exposure factors (FI). (ii) Independence assumption: IVs must be independent of confounding factors. (iii) Exclusivity hypothesis: IVs affect outcome only through FI and are not directly associated with outcome. Those with a significant association with phenotype (*p* <5*10^−8^) were screened out first. A total of 14 SNPs associated with FI were selected (*p* <5*10^−8^). SNPs that are similar in the genome have similar genetic effects, referred to as linkage disequilibrium, which may affect outcome effect values. To address this, we applied a threshold of r^2^ = 0.001 and a window size of 10,000 kb to remove the potential influence of linkage disequilibrium. The online tool PhenoScanner V2 [17,18] was used to further remove the effects of confounding factors, and SNPs associated with confounding factors such as obesity, adiposity, and smoking were excluded from our analysis. The strength of the instrumental variables is often assessed using the F-statistic in the exposure regression [19]. In our study, instrumental variables with an F-statistic <10 were considered weak and were excluded from further analysis.

### Statistical analysis

The FI on systemic atherosclerosis and lipids was evaluated using a two-sample MR analysis. The primary method employed to assess the causal association between FI and systemic atherosclerosis and lipids was the inverse variance weighted (IVW) method. A significance threshold of *p* <0.05 was set, and the results of causal associations were reported as odds ratio (OR) with 95% confidence interval (CI). The IVW method utilized a meta-analysis approach, combining Wald ratios for each genetic variant to generate combined estimates of the exposure’s effect on the outcomes, thereby providing consistent causal estimates [20]. In addition to the IVW method, other estimation methods such as the weighted median, simple median, and penalized weighted median were also employed. The simple median estimator is equivalent to a weighted median estimator with equal weights. While the simple median provides consistent causal effect estimates when at least 50% of the IVs are valid, the weighted median requires at least 50% of the weights to come from valid IVs [21]. Heterogeneity was assessed using Cochran’s Q test, and to evaluate pleiotropy, MR-Egger regression intercept and MR-PRESSO methods were utilized. Leave-one-out analysis was performed in the IVW method to determine if the results were driven by any specific SNP [22]. Reverse MR was used to estimate the causal association between systemic atherosclerosis and FI in the opposite direction. Multivariable MR, an expanded method of MR, was applied in situations where it is challenging to find IVs that are exclusively related to only one exposure, allowing for directed pleiotropy when IVs are associated with multiple exposures [23].

All statistical analyses were conducted using the "TwoSampleMR" and "MRPRESSO" packages in R version 4.3.0. A two-sided threshold of *p* <0.05 in the MR analysis was considered statistically significant.

## Results

After accounting for linkage disequilibrium effects, a total of 14 SNPs that showed an association with the FI at a significance level of *p* <5*10^−8^ were initially selected. However, further examination revealed that four of these SNPs (rs2071207, rs583514, rs1363103, and rs10891490) were associated with confounding factors such as weight, waist circumference, leg fat mass, smoking, and systolic blood pressure. Since this violated assumption (ii) of independence, these four SNPs were excluded from the IVs. As a result, a final set of 10 SNPs was defined as IVs to assess the causal relationship between the FI as the exposure factor and the outcome of interest (Table 2). These 10 SNPs were selected based on their relevance to the FI and their independence from confounding factors, meeting the requirements for conducting a robust Mendelian randomization analysis.

**Table 2:** Genetic variants significantly associated with the frailty index.

SNP	CHR:BP	Nearby gene	EA:OA	EAF	SE	beta	p	F-statistic
rs12739243	1:210302043	*SYT14*	T:C	0.78	0.004	0.024	1.28E-09	34.6
rs4952693	2:44151808	*LRPPRC*	T:C	0.37	0.003	-0.019	1.47E-08	29.5
rs82334	4:3225371	*HTT*	A:C	0.68	0.004	0.022	3.13E-10	36.9
rs9275160	6:32652620	*HLA-DQB1*	A:G	0.34	0.004	0.038	7.18E-28	113.6
rs2396766	7:114318071	*FOXP2*	A:G	0.47	0.003	0.020	1.22E-09	34.9
rs56299474	8:21992804	*REEP4*	A:C	0.17	0.004	0.024	3.94E-08	28.5
rs4146140	10:61885362	*ANK3*	T:C	0.38	0.003	-0.020	6.83E-09	33.0
rs3959554	15:41443924	*EXD1*, *INO80*	A:G	0.58	0.003	-0.019	1.74E-08	30.8
rs17612102	15:52264094	*LEO1*, *MAPK6*	T:C	0.41	0.003	-0.019	2.85E-08	30.6
rs8089807	18:39322639	*KC6*, *PIK3C3*	T:C	0.19	0.004	-0.025	6.50E-09	30.7

Chr, Chromosome; BP, Base pairs; EA, Effect allele; EAF, Effect allele frequency; OA, Other allele; SNP, Single nucleotide polymorphism.

### Genetically predicted FI and systemic atherosclerosis

**Fig 2:**
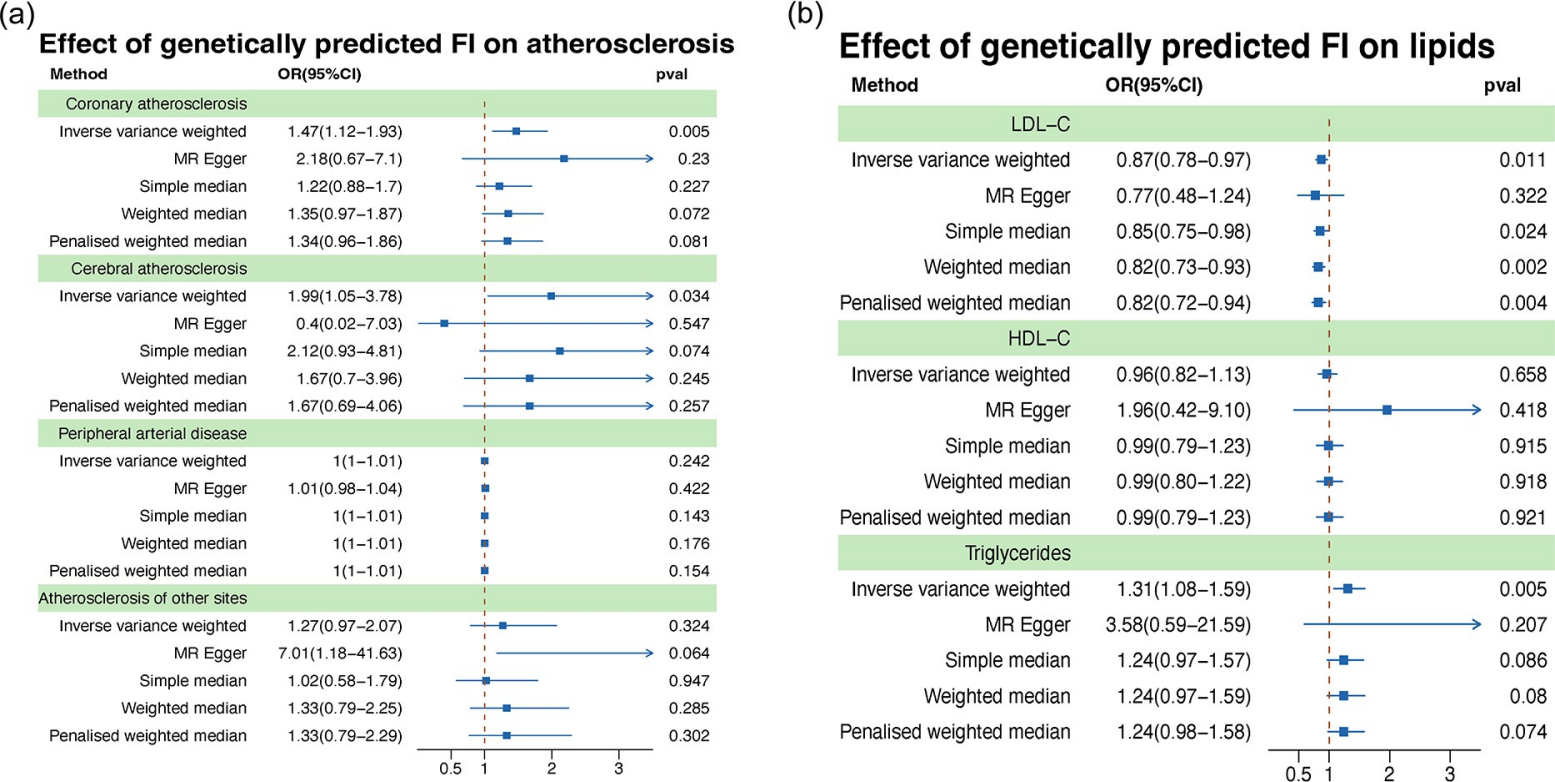
Genetically predicted FI on atherosclerosis and lipids. (a) Effect of genetically predicted FI on atherosclerosis, including coronary atherosclerosis, cerebral atherosclerosis, peripheral arterial disease, and atherosclerosis at other sites. (b) Effect of genetically predicted FI on lipids, including LDL-C, HDL-C, and triglycerides. MR, Mendelian randomization; FI, frailty index; LDL-C, LDL cholesterol; HDL-C, HDL cholesterol; OR, odds ratio; CI, confidence interval.

**Fig 3:**
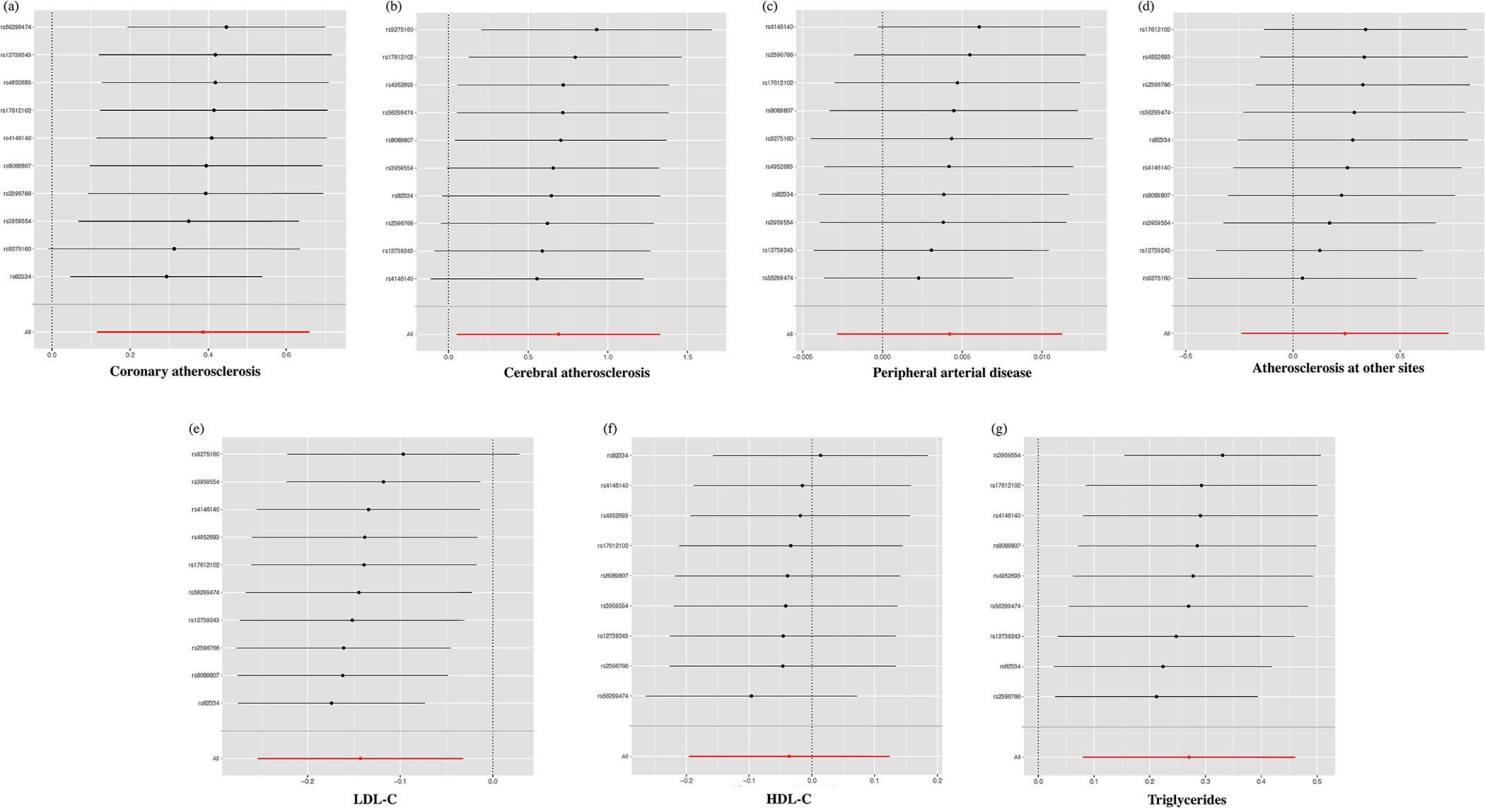
Leave-one-out analysis of the association between genetically predicted FI and risk of atherosclerosis and lipids. (a) coronary atherosclerosis; (b) cerebral atherosclerosis; (c) peripheral arterial disease; (d) atherosclerosis at other sites; (e) LDL-C; (f) HDL-C; (g) triglycerides. LDL-C, LDL cholesterol; HDL-C, HDL cholesterol.

We assessed the causal relationship between genetically predicted FI and systemic atherosclerosis, including coronary artery atherosclerosis, cerebral artery atherosclerosis, PAD, and atherosclerosis at other sites. The two-sample MR analysis using the IVW method revealed a positive association between genetically predicted FI and the risk of coronary artery atherosclerosis (OR = 1.47, 95% CI 1.12 to 1.93, *p* = 0.005) and cerebral artery atherosclerosis (OR = 1.99, 95% CI 1.05 to 3.78, *p* = 0.034). However, there was no significant causal relationship observed between genetically predicted FI and PAD (OR = 1, 95% CI 1 to 1.01, *p* = 0.242), or atherosclerosis at other sites (OR = 1.27, CI 0.79 to 2.07, *p* = 0.324) (Fig 2) (S1 File). Cochran’s Q test indicated that the estimates of IVs on the associations with coronary artery atherosclerosis and cerebral artery atherosclerosis did not exhibit heterogeneity (*p* >0.05). The MR-Egger regression intercept and MR-PRESSO methods provided no evidence of pleiotropy (*p* >0.05) (S2 File). Leave-one-out analysis indicated that the influence of FI on coronary artery atherosclerosis and cerebral artery atherosclerosis was not driven by a single SNP, indicating the overall stability of the results (Fig 3).

### Genetically predicted FI and lipids

We further evaluated the causal relationship between genetically predicted FI and lipids, including LDL-C, HDL-C, and triglycerides. The two-sample MR analysis using the IVW method revealed a positive association between genetically predicted FI and triglyceride levels (OR = 1.31, CI 1.08 to 1.59, *p* = 0.005). There was a negative association between genetically predicted FI and LDL-C levels (OR = 0.87, CI 0.78 to 0.97, *p* = 0.011). However, there was no significant association observed between genetically predicted FI and HDL-C levels (OR = 0.96, CI 0.82 to 1.13, *p* = 0.658) (Fig 2) (S3 File). Cochran’s Q test indicated that the estimates of IVs on LDL-C, HDL-C, and triglyceride levels did not exhibit heterogeneity (*p* >0.05). The MR-Egger regression intercept and MR-PRESSO methods showed no evidence of pleiotropy (*p* >0.05) (S2 File). Leave-one-out analysis indicated that the influence of FI on LDL-C and triglyceride levels was not driven by a single SNP (Fig 3).

### Reverse MR and multivariate MR

To investigate the causal relationship between systemic atherosclerosis and FI, we performed a reverse MR analysis. The results revealed a significant causal association between atherosclerosis at different anatomical locations and FI. Specifically, coronary artery atherosclerosis (OR = 1.06, 95% CI 1.04 to 1.07, *p* <0.001), cerebral artery atherosclerosis (OR = 1.03, 95% CI 1.01 to 1.05, *p* <0.001), PAD (*p* <0.001), and atherosclerosis at other sites (OR = 1.10, 95% CI 1.08 to 1.12, *p* <0.001) exhibited a clear causal association with FI (S4 File).

Furthermore, we employed multivariable MR analysis to assess the causal relationship between lipids and FI. The results indicated that LDL-C (OR = 1.08, CI 1.04 to 1.12, *p* <0.001), HDL-C (OR = 0.97, 95% CI 0.96 to 0.98, *p* <0.001), and triglycerides (OR = 0.96, CI 0.93 to 0.99, *p* = 0.008) had a significant causal association with FI (S5 File). Among these lipids, LDL-C and HDL-C showed a stronger correlation with FI than triglycerides.

## Discussion

Our results indicate that the genetically predicted FI is associated with a higher risk of coronary artery atherosclerosis and cerebral artery atherosclerosis, while no significant associations were found with PAD and atherosclerosis at other sites. Moreover, genetically predicted FI is positively correlated with triglyceride levels, negatively correlated with LDL-C levels, and not significantly associated with HDL-C levels.

One of the pressing challenges in public health management is the global phenomenon of population aging [24]. As the number of frail individuals continues to rise, there are growing concerns about the decline in quality of life and the substantial economic burden associated with cardiovascular diseases. In the past decade, there has been increasing interest in understanding the relationship between frailty and cardiovascular diseases. A prospective cohort study [25] identified frailty as a significant risk factor for adverse cardiovascular events. Even after accounting for baseline factors such as age, sex, and ethnicity, frail individuals exhibited notably higher rates of cardiovascular events, including coronary heart disease, stroke, and PAD, than their non-frail counterparts. This highlights the importance of addressing frailty as a means of preventing and potentially reversing the occurrence of cardiovascular events. Priority should be given to strategies that target frail populations for screening for coronary atherosclerosis and cerebral atherosclerosis. By focusing on interventions that target frailty, it may be possible to have a positive impact on reducing the burden of cardiovascular diseases.

The FI is a well-established and validated instrument that relies on a questionnaire survey assessing functional deficits [1]. In frail individuals, the underlying pathological mechanisms for cardiovascular events are related to atherosclerosis and abnormal lipids. Observational studies have consistently indicated a connection between FI and cardiovascular events or subclinical atherosclerosis. Several cross-sectional analyses [26–28] have demonstrated that individuals with higher FI scores or in a prefrail state are more susceptible to cardiovascular diseases. Moreover, elevated FI scores have been associated with increased cardiovascular mortality rates [29]. A longitudinal study conducted over a 16-year period found that for every 0.1 increase in FI, the risk of all-cause mortality increased by 4% and the risk of cardiovascular disease-related mortality increased by 3–5% [30].

In patients with acute stroke, frailty can be exacerbated, as confirmed by a meta-analysis. Although certain studies may carry a risk of bias, frailty is commonly observed in individuals with acute stroke, regardless of the specific measurement method used. Two meta-analyses [9,31] have demonstrated a significantly higher prevalence of frailty among individuals with PAD. Additionally, research has shown an independent association between subclinical cardiovascular diseases, such as carotid artery atherosclerosis and femoral artery sclerosis, and frailty, irrespective of the presence of coronary heart disease, stroke, or myocardial infarction [32].

The relationship between PAD and frailty remains a topic of debate. While frailty is often observed in individuals with PAD, observational studies have not definitively established a bidirectional causal relationship between PAD and frailty. A study conducted by Brutto suggested that there is no independent correlation between frailty and large artery atherosclerosis in the peripheral vascular bed [33]. This could be due to the onset of frailty affecting various anatomical districts. In fact, frailty is a state of multi-organ fictional decline resulting from the disruption of the balance among multiple organs, which are unable to support each other [34]. Vital organs such as the heart and brain, which are supplied by major blood vessels, are more likely to be involved. In other words, individuals with a high FI have an increased risk of developing cardiovascular diseases affecting the heart and brain but not necessarily an increased risk of peripheral artery atherosclerosis or atherosclerosis in other arteries. This may explain the varying relationship between frailty and different types of atherosclerosis.

Blood lipids, such as triglycerides and LDL-C, play a significant role in the development of atherosclerosis, and research indicates that lowering these lipid levels can reduce the risk of cardiovascular diseases [35]. A Bayesian network analysis conducted on community-dwelling older people suggested that HDL-C levels may be associated with malnutrition and can have an impact on frailty status [36]. This finding aligns with the results of our reverse MR analysis. It is worth noting that HDL-C levels are more closely associated with age, with LDL-C levels declining further in older individuals [37]. However, frailty is not solely determined by age and does not exhibit a clear positive causal relationship with HDL-C levels. This discrepancy may be attributed to the complex interplay of multiple factors influencing frailty beyond chronological age.

Our study provides a systematic evaluation of the causal relationship between frailty and atherosclerosis occurring in different parts of the body, incorporating the association estimates of blood lipids. We utilized the latest GWAS data with an adequate sample size to ensure reliable results. However, our study still has limitations. First, the potential bias in MR analysis due to population and age structure should be considered. The GWAS data used in our study primarily originated from European, Swedish and Finnish populations, predominantly consisting of older adults and females [25], which may limit the generalizability of our findings to other populations. Second, despite our efforts to address pleiotropic effects, such as removing linkage disequilibrium effects and weak instrument variables and applying methods such as MR-Egger regression intercept and MR-PRESSO to account for horizontal pleiotropy, these methods have their inherent limitations. Although we utilized the PhenoScanner V2 tool to control for confounders, it is challenging to eliminate the influence of horizontal pleiotropy. Third, our assessment of blood lipids focused on LDL-C, HDL-C, and triglycerides, while other lipid measures were not included in our analysis. Future research should explore additional lipid indicators, such as apolipoprotein A1 and apolipoprotein B, to gain a more comprehensive understanding of lipid metabolism in relation to frailty and atherosclerosis.

Overall, our study highlights the bidirectional causal relationship between FI and systemic atherosclerosis, as well as the association between FI and lipids, taking into account the specific locations of arterial atherosclerosis. These findings contribute to a better understanding of the complex interplay between frailty, atherosclerosis, and lipid metabolism.

## Conclusion

Our study elucidated that genetically predicted FI is associated with the risk of coronary atherosclerosis and cerebral atherosclerosis by the MR analysis method, and they have a bidirectional causal relationship. Moreover, genetically predicted FI was causally associated with triglyceride and LDL-C levels. Further understanding of this association is crucial for optimizing medical practice and care models specifically tailored to frail populations.

## Supporting information

S1 File Effect of genetically predicted FI on atherosclerosis. FI, Frailty Index; MR, Mendelian randomization; OR, Odds ratio; CI, Confidence interval.
(PDF)

S2 File Heterogeneity and pleiotropy analysis. FI, Frailty Index; MR, Mendelian randomization; IVW, Inverse variance weighted; LDL-C, LDL cholesterol; HDL-C, HDL cholesterol.
(PDF)

S3 File Effect of genetically predicted FI on lipids. FI, Frailty Index; MR, Mendelian randomization; LDL-C, LDL cholesterol; HDL-C, HDL cholesterol; OR, Odds ratio; CI, Confidence interval.
(PDF)

S4 File Reverse MR of genetically predicted effects of atherosclerosis on FI. FI, Frailty Index; MR, Mendelian randomization; IVs, Instrumental variables; OR, Odds ratio; CI, Confidence interval.
(PDF)

S5 File Multivariate MR of the effect of genetically predicted lipids on FI. FI, Frailty Index; MR, Mendelian randomization; IVs, Instrumental variables; LDL-C, LDL cholesterol; HDL-C, HDL cholesterol; OR, Odds ratio; CI, Confidence interval.
(PDF)

## Abbreviations

MR: Mendelian Randomization
FI: Frailty Index
GWAS: Genome-Wide Association Studies
PAD: Peripheral Arterial Disease
IVW: Inverse Variance Weighted
SNPs: Single Nucleotide Polymorphisms
IVs: Instrumental Variables
LDL-C: LDL Cholesterol
HDL-C: HDL Cholesterol
IEU: Integrative Epidemiology Unit
OR: Odds Ratio
CI: Confidence Interval
