# Supplementary material for: Frailty and risk of systemic atherosclerosis: A bidirectional Mendelian randomization study
Source: PLoS One. 2024 May 23;19(5):e0304300. doi: 10.1371/journal.pone.0304300 (PMC11115302; doi:10.1371/journal.pone.0304300)
Supplement: S1 File — FI, Frailty Index; MR, Mendelian randomization; OR, Odds ratio; CI, Confidence interval. (PDF) [file pone.0304300.s001.pdf]

S1 File 1: Effect of genetically predicted FI on atherosclerosis

Method	OR	OR_low_95%CI	OR_up_95%CI	p.value	OR(95%CI)
**Coronary atherosclerosis**					
Inverse variance weighted	1.47	1.12	1.93	0.005	1.47(1.12-1.93)
MR Egger	2.18	0.67	7.1	0.23	2.18(0.67-7.1)
Simple median	1.22	0.88	1.7	0.227	1.22(0.88-1.7)
Weighted median	1.35	0.97	1.87	0.072	1.35(0.97-1.87)
Penalised weighted median	1.34	0.96	1.86	0.081	1.34(0.96-1.86)
**Cerebral atherosclerosis**					
Inverse variance weighted	1.99	1.05	3.78	0.034	1.99(1.05-3.78)
MR Egger	0.4	0.02	7.03	0.547	0.4(0.02-7.03)
Simple median	2.12	0.93	4.81	0.074	2.12(0.93-4.81)
Weighted median	1.67	0.7	3.96	0.245	1.67(0.7-3.96)
Penalised weighted median	1.67	0.69	4.06	0.257	1.67(0.69-4.06)
**Peripheral arterial disease**					
Inverse variance weighted	1	1	1.01	0.242	1(1-1.01)
MR Egger	1.01	0.98	1.04	0.422	1.01(0.98-1.04)
Simple median	1	1	1.01	0.143	1(1-1.01)
Weighted median	1	1	1.01	0.176	1(1-1.01)
Penalised weighted median	1	1	1.01	0.154	1(1-1.01)
**Atherosclerosis at other sites**					
Inverse variance weighted	1.27	0.79	2.07	0.324	1.27(0.97-2.07)
MR Egger	7.01	1.18	41.63	0.064	7.01(1.18-41.63)
Simple median	1.02	0.58	1.79	0.947	1.02(0.58-1.79)
Weighted median	1.33	0.79	2.25	0.285	1.33(0.79-2.25)
Penalised weighted median	1.33	0.77	2.29	0.302	1.33(0.79-2.29)

Abbreviations: FI, Frailty Index; MR, Mendelian randomization; OR, Odds ratio; CI, Confidence interval
