# Supplementary material for: Frailty and risk of systemic atherosclerosis: A bidirectional Mendelian randomization study
Source: PLoS One. 2024 May 23;19(5):e0304300. doi: 10.1371/journal.pone.0304300 (PMC11115302; doi:10.1371/journal.pone.0304300)
Supplement: S2 File — FI, Frailty Index; MR, Mendelian randomization; IVW, Inverse variance weighted; LDL-C, LDL cholesterol; HDL-C, HDL cholesterol. (PDF) [file pone.0304300.s002.pdf]

S2 File 2: Heterogeneity and pleiotropy analysis

Exposure	Outcome	IVW Q statistic (P-value)	MR-Egger intercept (P-value)	MR-PRESSO P-value	
FI	Coronary atherosclerosis	12.36(0.194)	-0.009(0.519)	0.230	
FI	Cerebral atherosclerosis	5.94(0.746)	0.038(0.292)	0.710	
FI	Peripheral arterial disease	28.87(<0.001)	<0.001(0.57)	0.939	
FI	Atherosclerosis at other sites	16.54(0.056)	-0.041(0.089)	0.063	
FI	LDL-C	13.85(0.128)	0.003(0.643)	0.148	
FI	HDL-C	7.85(0.448)	-0.015(0.393)	0.412	
FI	Triglycerides	10.45(0.235)	-0.021(0.308)	0.259	

Abbreviations: FI, Frailty Index; MR, Mendelian randomization; IVW, Inverse variance weighted; LDL-C, LDL cholesterol; HDL-C, HDL choles
