# Supplementary material for: Frailty and risk of systemic atherosclerosis: A bidirectional Mendelian randomization study
Source: PLoS One. 2024 May 23;19(5):e0304300. doi: 10.1371/journal.pone.0304300 (PMC11115302; doi:10.1371/journal.pone.0304300)
Supplement: S5 File — FI, Frailty Index; MR, Mendelian randomization; IVs, Instrumental variables; LDL-C, LDL cholesterol; HDL-C, HDL cholesterol; OR, Odds ratio; CI, Confidence interval. (PDF) [file pone.0304300.s005.pdf]

S5 File 5: Multivariate MR of the effect of genetically predicted lipids on FI									
Study number	Exposure	Outcome	IVs	beta	se	p.value	OR	OR_low_95%CI	OR_up_95%CI
ieu-b-4844	HDL-C	FI	54	-0.029670496	0.005704687	1.98E-07	0.97	0.96	0.98
ieu-b-4850	Triglycerides	FI	33	-0.044116715	0.016794377	8.62E-03	0.96	0.93	0.99
ieu-b-5089	LDL-C	FI	59	0.077495681	0.019280031	5.83E-05	1.08	1.04	1.12
Abbreviations: FI, Frailty Index; MR, Mendelian randomization; IVs, Instrumental variables; LDL-C, LDL cholesterol; HDL-C, HDL cholesterol; OR, Odds ratio; CI, Confidence interval									
