# Supplementary material for: Frailty and risk of systemic atherosclerosis: A bidirectional Mendelian randomization study
Source: PLoS One. 2024 May 23;19(5):e0304300. doi: 10.1371/journal.pone.0304300 (PMC11115302; doi:10.1371/journal.pone.0304300)
Supplement: S3 File — FI, Frailty Index; MR, Mendelian randomization; LDL-C, LDL cholesterol; HDL-C, HDL cholesterol; OR, Odds ratio; CI, Confidence interval. (PDF) [file pone.0304300.s003.pdf]

S3 File 3: Effect of genetically predicted FI on lipids					
**Method**	**OR**	**OR_low_95%CI**	**OR_up_95%CI**	**p.value**	**OR(95%CI)**
**LDL-C**					
Inverse variance weighted	0.87	0.78	0.97	0.011	0.87(0.78-0.97)
MR Egger	0.77	0.48	1.24	0.322	0.77(0.48-1.24)
Simple median	0.85	0.75	0.98	0.024	0.85(0.75-0.98)
Weighted median	0.82	0.73	0.93	0.002	0.82(0.73-0.93)
Penalised weighted median	0.82	0.72	0.94	0.004	0.82(0.72-0.94)
**HDL-C**					
Inverse variance weighted	0.96	0.82	1.13	0.658	0.96(0.82-1.13)
MR Egger	1.96	0.42	9.1	0.418	1.96(0.42-9.10)
Simple median	0.99	0.79	1.23	0.915	0.99(0.79-1.23)
Weighted median	0.99	0.8	1.22	0.918	0.99(0.80-1.22)
Penalised weighted median	0.99	0.79	1.23	0.921	0.99(0.79-1.23)
**Triglycerides**					
Inverse variance weighted	1.31	1.08	1.59	0.005	1.31(1.08-1.59)
MR Egger	3.58	0.59	21.59	0.207	3.58(0.59-21.59)
Simple median	1.24	0.97	1.57	0.086	1.24(0.97-1.57)
Weighted median	1.24	0.97	1.59	0.08	1.24(0.97-1.59)
Penalised weighted median	1.24	0.98	1.58	0.074	1.24(0.98-1.58)

Abbreviations: FI, Frailty Index; MR, Mendelian randomization; LDL-C, LDL cholesterol; HDL-C, HDL cholesterol; OR, Odds ratio; CI, Confidence interval
