# Supplementary material for: Frailty and risk of systemic atherosclerosis: A bidirectional Mendelian randomization study
Source: PLoS One. 2024 May 23;19(5):e0304300. doi: 10.1371/journal.pone.0304300 (PMC11115302; doi:10.1371/journal.pone.0304300)
Supplement: S4 File — FI, Frailty Index; MR, Mendelian randomization; IVs, Instrumental variables; OR, Odds ratio; CI, Confidence interval. (PDF) [file pone.0304300.s004.pdf]

S4 File 4: Reverse MR of genetically predicted effects of atherosclerosis on FI									
Method	Exposure	Outcome	IVs	beta	se	p.val	OR	OR_low_95%CI	OR_up_95%CI
Inverse variance weighted	**Coronary atherosclerosis**	FI	56	0.06	0.01	3.34E-13	1.06	1.04	1.07
MR Egger	**Coronary atherosclerosis**	FI	56	0.10	0.01	5.72E-11	1.1	1.08	1.13
Simple median	**Coronary atherosclerosis**	FI	56	0.06	0.01	3.39E-14	1.06	1.05	1.08
Weighted median	**Coronary atherosclerosis**	FI	56	0.02	0.01	5.64E-03	1.02	1.01	1.04
Penalised weighted median	**Coronary atherosclerosis**	FI	56	0.03	0.01	2.51E-04	1.03	1.01	1.05
Inverse variance weighted	**Cerebral atherosclerosis**	FI	4	0.03	0.01	7.52E-04	1.03	1.01	1.05
MR Egger	**Cerebral atherosclerosis**	FI	4	-0.02	0.04	6.38E-01	0.98	0.91	1.06
Simple median	**Cerebral atherosclerosis**	FI	4	0.03	0.01	5.77E-03	1.04	1.01	1.06
Weighted median	**Cerebral atherosclerosis**	FI	4	0.03	0.01	1.68E-03	1.03	1.01	1.06
Penalised weighted median	**Cerebral atherosclerosis**	FI	4	0.03	0.01	2.70E-03	1.03	1.01	1.06
Inverse variance weighted	**Peripheral arterial disease**	FI	2	8.24	1.33	6.41E-10	3772.87	276.89	51408.39
Inverse variance weighted	**Atherosclerosis at other sites**	FI	10	0.09	0.01	3.92E-27	1.1	1.08	1.12
MR Egger	**Atherosclerosis at other sites**	FI	10	0.11	0.02	2.16E-04	1.11	1.08	1.15
Simple median	**Atherosclerosis at other sites**	FI	10	0.08	0.02	1.98E-07	1.08	1.05	1.12
Weighted median	**Atherosclerosis at other sites**	FI	10	0.06	0.01	4.22E-07	1.06	1.03	1.08
Penalised weighted median	**Atherosclerosis at other sites**	FI	10	0.06	0.01	2.13E-07	1.06	1.04	1.08
Abbreviations: FI, Frailty Index; MR, Mendelian randomization; IVs, Instrumental variables; OR, Odds ratio; CI, Confidence interval									
